# Xanthohumol suppresses glioblastoma via modulation of Hexokinase 2 -mediated glycolysis

**DOI:** 10.7150/jca.33045

**Published:** 2020-04-06

**Authors:** Jian Yuan, Gang Peng, Gelei Xiao, Zhuanyi Yang, Jun Huang, Qing Liu, Zhiquan Yang, Dingyang Liu

**Affiliations:** 1 Department of Neurosurgery, Xiangya Hospital, Central South University, 87 Xiangya Road, Changsha, Hunan 410008, China.; 2 The Institute of Skull Base Surgery and Neuro-oncology at Hunan, 87 Xiangya Road, Changsha, Hunan, 410008, China.

**Keywords:** glioblastoma, xanthohumol, Hexokinases II, c-Myc, glycolysis

## Abstract

Deregulation of aerobic glycolysis is a common phenomenon in human cancers, including glioblastoma (GBM). In the present study, we demonstrated that the natural compound xanthohumol has a profound anti-tumor effect on GBM via direct inhibition of glycolysis. Xanthohumol suppressed cell proliferation and colony formation of GBM cells, and significantly impaired glucose metabolism via inhibiting Hexokinase 2 (HK2) expression. We demonstrated that down-regulation of c-Myc was required for xanthohumol-induced decrease of HK2. Xanthohumol destabilization of c-Myc, and promoted FBW7-mediated ubiquitination of c-Myc. Xanthohumol attenuated Akt activity and inhibited the activation of GSK3β, resulted in c-Myc degradation. Overexpression of Myr-Akt1 significantly rescued xanthohumol-mediated c-Myc inhibition and glycolysis suppression. Finally, the xanthohumol-mediated down-regulation of the PI3-K/Akt-GSK3beta-FBW7 signaling axis promoted the destabilization of c-Myc. Finally, the animal results demonstrated that xanthohumol substantially inhibited tumor growth *in vivo*. Collectively, xanthohumol appears to be a promising new anti-tumor agent with the therapeutic potential for GBM.

## Introduction

Glioblastoma (GBM) is one of the most common and aggressive brain tumors in human adults.

Despite the combined therapeutic approach, including aggressive surgery, adjuvant postoperative radio/chemotherapy, the 5-year survival rate of glioblastoma patients is less than 5% 1-4. Resistance to traditionally chemo/radiotherapy or targeted therapy is the crucial factor to result in therapy failure in glioblastoma 5-7. Recently, human cancer tissues based sequencing has revealed that the mutation of tumor suppressor genes, or/and hyperactivation of oncogenes, was involved in glioblastoma tumorigenesis 8-10. However, the relationship between alterations of gene expression profile and GBM tumorigenesis is still unclear. Elucidating the underlying mechanism and development of novel approaches for diagnosis and therapy is still an urgent demand for clinic GBM treatment.

An accumulation of evidence reveals that xanthohumol (XN), a prenylated chalcone extracted from hop plant Humulus lupulus L. (Cannabaceae), has the potential for cancer prevention and therapy 11. Experimental studies from laboratory and epidemiological investigations and have demonstrated that xanthohumol suppresses a panel of human malignancies, including lung 12, ovarian 13, breast 14, pancreatic 15, prostate 16 and liver 17 cancer. Xanthohumol reportedly processes its anti-tumor effects via inhibition of various signaling pathways, such as disruption of the activation of transcription factors, suppression of multiple protein kinases, and regulation of the expression of genes which related to cell proliferation, angiogenesis, and apoptosis 15, 18, 19. Nonetheless, the anti-tumor activity of xanthohumol in GBM and its potential targets were not clear.

Recently evidence suggested that most human cancer cells preferentially take glycolysis to rapidly generate ATP even in the presence of oxygen 20. Hexokinase 2, also known as HK2 is an enzyme which in humans is encoded by the HK2 gene on chromosome 2. Hexokinases phosphorylate glucose to produce glucose-6-phosphate (G6P), the first rate-limiting step of glycolysis 21, 22. HK-2 was suggested to be highly expressed in multiple types of human cancer. Overexpression of HK2 predicts poor prognosis in human non-small cell lung cancer 23-25, breast cancer 26, and hepatocellular carcinoma 27.Previous studies have demonstrated that GBM cells prefer to acquire energy from glycolysis instead of oxidative phosphorylation, and suppression of glycolysis is proposed to be a promising treatment option for human GBM 28-30. Here, we found that the degradation of c-Myc played a crucial role in xanthohumol-induced down-regulation of glycolysis and HK2 in GBM cells. Importantly, xanthohumol inhibited Akt signaling pathway and promoted FBW7-mediated ubiquitination of c-Myc. This study provides novel insights into the molecular mechanisms of xanthohumol-induced antitumor effect, and targeting HK2 might be a new strategy for GBM treatment.

## Methods

**Cell Culture and Transfection.** The human U87-MG, T98G and LN229 glioblastoma cell lines were purchased from American Type Culture Collection (ATCC, Manassas, VA), and cultured according to the ATCC protocols. For transient transfection, the Lipofectamine™ 2000 Transfection Reagent (Waltham, MA) was used following the manufacturer's protocols. **Reagents and Antibodies.** Xanthohumol (>99%) and Irinotecan (>99%) were from Selleck Chemicals (Houston, TX). Cell culture media, including the supplements were purchased from Invitrogen (Grand Island, NY). Antibodies against p-Akt (#4060), HK2 (#2867), Akt (#4691), p-S6 (#4858, Akt1 (#75692), S6 (#2317), cleaved-caspase 3 (#9664), VDAC1 (#4866), cleaved-caspase 9 (#9505), cytochrome C (#4280) and cleaved-PARP (#5625) were purchased from Cell Signaling Technology, Inc. (Beverly, MA). The β-actin (A5316, IB: 1:10000) antibody was obtained from Sigma-Aldrich, and α-Tubulin (SC-5286, IB: 1:5000) antibody was from Santa Cruz (Dallas, TX). Anti-Ki67 (ab16667) for IHC was from Abcam (Cambridge, UK).

**MTS Assay.** Human GBM cells were seeded into 96-well plates at the concentration of 3×10^3^/well. Cells were treated with DMSO control, xanthohumol, or irinotecan for various time points as indicated. Cell proliferation was examined via MTS assay (Promega, Madison, WI) following the protocol provided.

**Anchorage-independent Growth.** The colony formation assay was performed as previously described 31. Briefly**,** Human GBM cells were suspended at the concentration of 8,000 cells/ml in 1 ml of 0.3% agar with 10% FBS in Eagle's basal medium, and various doses of xanthohumol or irinotecan. The mixture was overlaid into the 0.6% agar base contained six-well plates, the cultures were maintained for 2 weeks. The colonies in soft agar were counted using the microscope with the Image-Pro Plus software program (Media Cybernetics, Silver Spring, MD).

**Measurement of Glucose Uptake and Lactate Production.** Human GBM cells were seeded into 6-well plate at the concentration of 1×10^6^/well. The cells were cultured for 6 h, then the fresh medium with different concentrations of xanthohumol was added, and the cells were cultured for an additional 8 h. Glucose uptake and lactate production levels were tested at the Clinical Biochemical Laboratory of Xiangya Hospital (Changsha, China). Protein concentration was used for normalization the relative glucose consumption and lactate production rate.

**Immunoblotting and Immunoprecipitation.** Protein preparation and Western blotting were performed as the methods described previously 32. Briefly, whole-cell lysates were extracted with RIPA buffer (ThermoFisher, Waltham, MA). The lysates were concentration using the BCA protein kit (ThermoFisher). Protein samples were separated by SDS-PAGE, followed by transfer onto PVDF membranes and antibody incubation. The protein band was visualized via the enhanced chemiluminescence detection kit (ThermoFisher). The Pierce™ IP Lysis Buffer (ThermoFisher) was used for immunoprecipitation. The lysates were precleared with agarose A/G beads followed by incubation with antibody and fresh agarose A/G beads. The beads were washed and mixed with 5×SDS loading buffer, boiled, and subjected to western blot analysis.

**Isolation of Mitochondrial Fractions.** Following compound treatment, the cells were harvested and centrifuged at 800×g for 2 min at 4°C. The Mitochondrial Fractions were isolated using the Mitochondria Isolation Kit (#89874, Thermo Fisher) according to the protocol provided.

**Flow Cytometry.** Following compound treatment, the cells were harvested and centrifuged at 800×g for 5 min. The cell pellets were suspended at the concentration of 1×10^6^ cells/ml, 5 µl Propidium Iodide and Annexin V were added to the cell suspension. The mixture was incubated at room temperature for 15 min in the dark. Apoptosis cells were quantified using a FACSort Flow Cytometer (BD, San Jose, CA, USA).

**Xenograft Mouse Growth.** All of the animal studies were approved by the Animal Ethics Committee of Xiangya Hospital, Hunan, China. U87-MG or LN229 cells (2 × 10^6^) in 100 μL DMEM medium were inoculated s.c. into the right flank of 6-week-old female athymic nude mice. After tumor volume reached around 100 mm^3^, the xanthohumol-treated group was administered xanthohumol at a dose of 10 mg/kg by i.p. injection of every three days, or irinotecan, 5 mg/kg daily every 3 days. The control-treated group was administered vehicle control. The tumor volume and body weight were determined by vernier caliper every three days.

**Immunohistochemical Staining.** The IHC staining was performed as previously described 33. Briefly, tissue sections were baked for 2 h at 60°C, followed by deparaffinized and rehydrated. Then the slides were boiled in sodium citrate buffer (10 mM, pH 6.0) for 10 min, followed by immersing into 3% H_2_O_2_ for 10 min. The slides were blocked with 50% goat serum albumin in 1×PBS for 1 h at room temperature. The slides were incubated with the primary antibody in a humidified chamber overnight. After incubation with the secondary antibody at room temperature for 1 h, the slides were stained using the Vectastain Elite ABC kit.

**Statistical Analysis.** The quantitative data are expressed as mean values ± S.D of 3 independent experiments. Student's *t* test or Mann-Whitney *U*-test was used for determining the significant differences. A probability value of *p* < 0.05 was used as the criterion for statistical significance.

## Results

**Figure 1:**
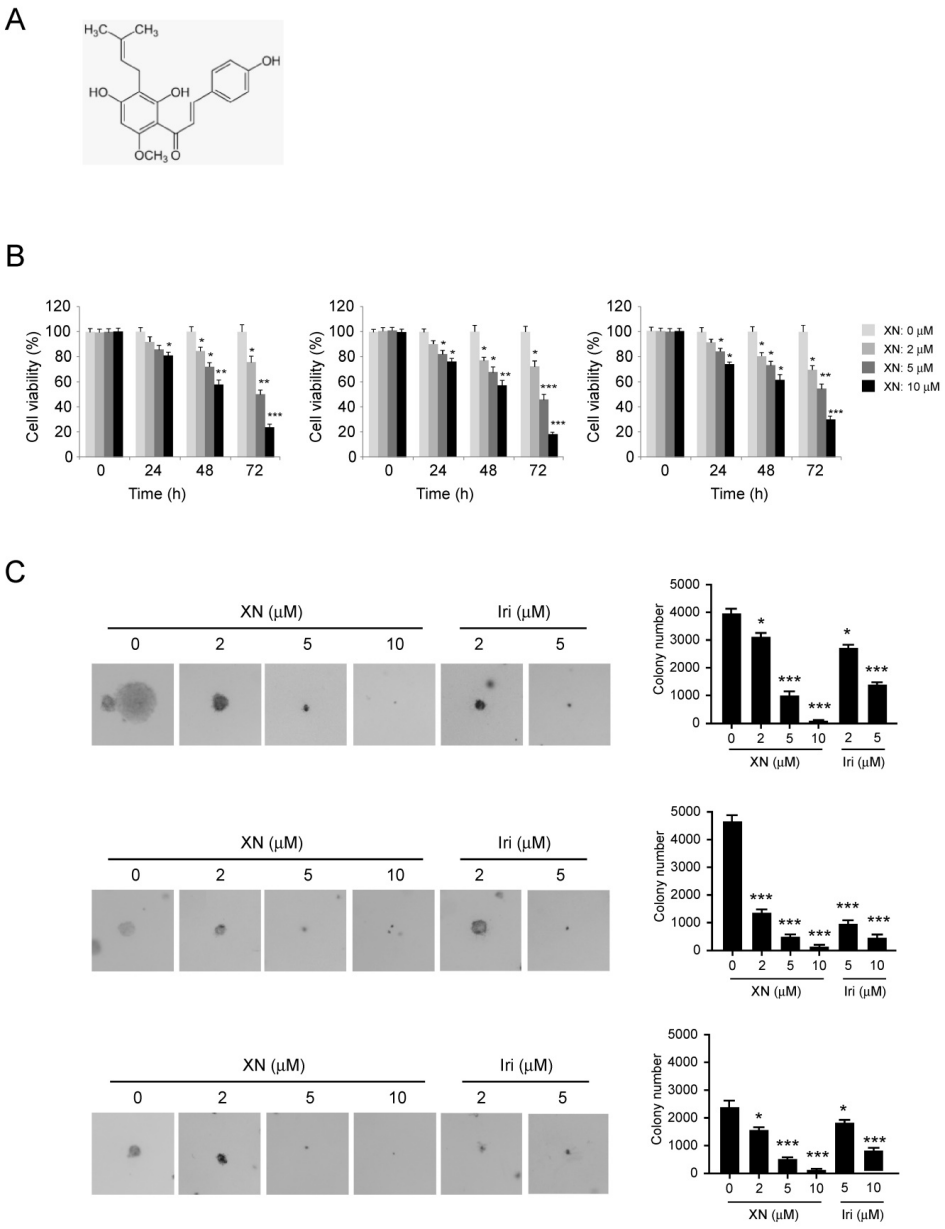
Inhibitory effects of xanthohumol on the growth of GBM cells. A, Chemical structure of Xanthohumol. B, Xanthohumol inhibits anchorage-dependent growth in a panel of human GBM cells, including U87 (left), T98G (middle), and LN229 (right). Cell proliferation assay was performed as described in the “Material and Methods”. Data shown are the proliferation ability of human GBM cells treated with different concentrations of xanthohumol compared with the DMSO-treated group, asterisk, significant suppression (*p<0.05, **p<0.01, ***p<0.001) of proliferation by Xanthohumol. C. The soft agar colony formation assay was performed as described in the “Material and Methods”. Data shown are the colony formation ability of U87 (up), T98G (middle), and LN229 (bottom) cells treated with different concentrations of Xanthohumol or irinotecan. The average colony number was calculated from three separate experiments. Iri, Irinotecan. Asterisk, significant suppression (*p<0.05, **p<0.01, ***p<0.001) of colony formation by xanthohumol compared with DMSO-treated group.

**Figure 2:**
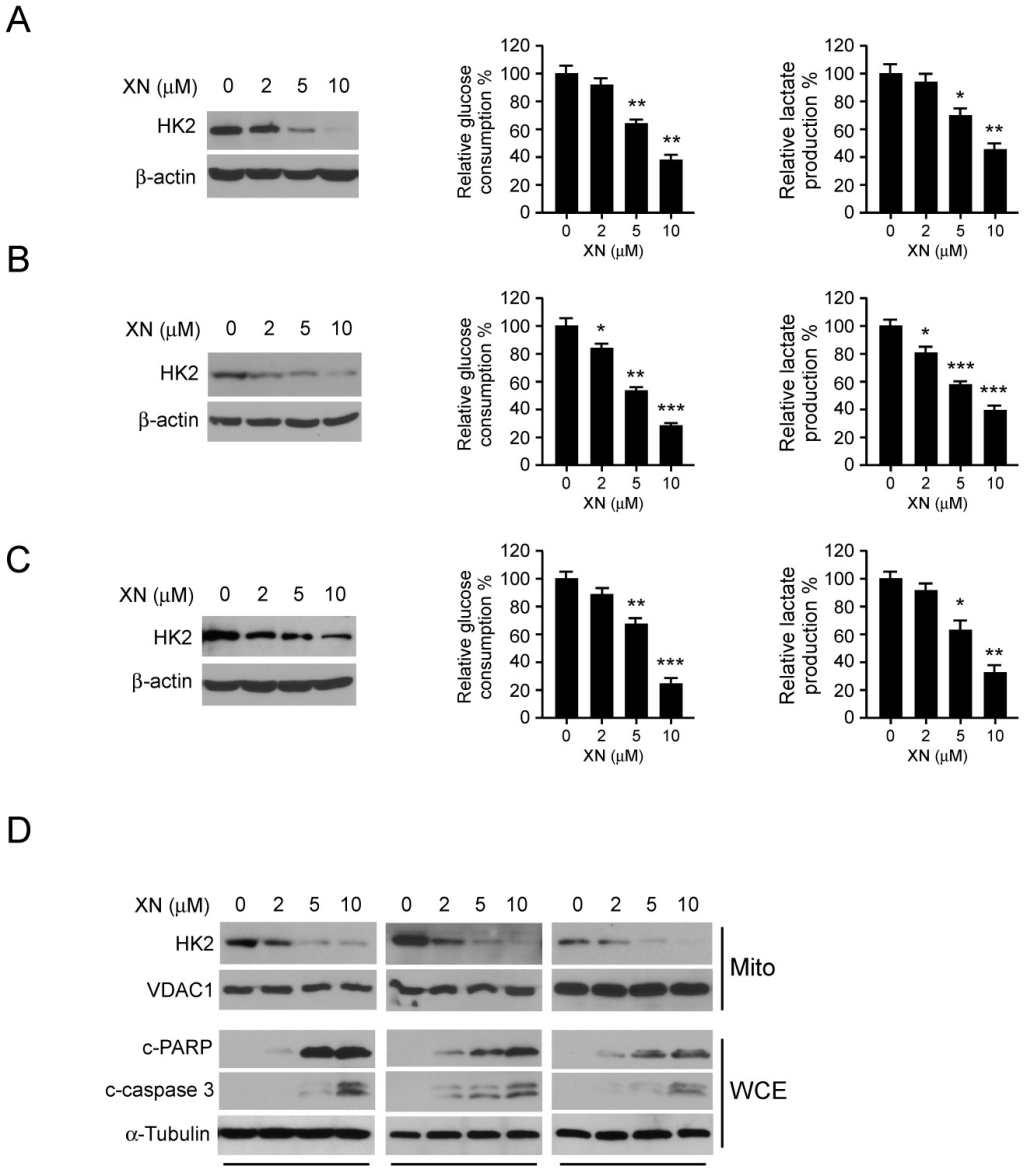
Xanthohumol suppresses glycolysis in GBM cells. GBM cells, including U87 (A), T98G (B) and LN229 (C) were treated with xanthohumol as indicated for 24 h. Western blotting was performed to determine the protein level of HK2, β-actin was used as a loading control (A, B and C, left). The levels of glucose consumption (A, B and C, middle) and lactate production (A, B, and C, right) were examined in these cells. The asterisk, significant suppression (*p<0.05, **p<0.01, ***p<0.001) of glycolysis by xanthohumol compared with DMSO-treated group. D, xanthohumol inhibits HK2 localization on mitochondria and up-regulates apoptosis. Human GBM cells, including U87 (left), T98G (middle) and LN229 (right) cells were treated with xanthohumol for 24 h, the mitochondrial fractions and whole-cell extracts were isolated, western blot was conducted to detect target proteins as indicated.

**Xanthohumol suppresses cell growth of glioblastoma cells.** The natural compound xanthohumol (Figure 1A, MW. 354.39 g/mol) has shown potential chemotherapy activities against human cancers. Here, we showed that xanthohumol significantly inhibited the proliferation of human GBM cells in a dose- and time-dependent manner, including U87 (Figure 1B, left), T98G (Figure 1B, middle) and LN229 (Figure 1B, right). We compared the antitumor effect of xanthohumol with the chemotherapeutic agent irinotecan. Our results showed that both xanthohumol and irinotecan suppressed tumor cell viability. Irinotecan exhibited a much stronger effect at the same concentration of 5 μM for 72 h treatment (Supplementary Figure 1A-C). We further tested the inhibitory effect of xanthohumol and irinotecan on colony formation of U87 (Figure 1C, left), T98G (Figure 1C, middle) and LN229 (Figure 1C, right) cells. Results showed that xanthohumol dramatically inhibited colony formation of GBM cells at 2 μM, and 10 μM xanthohumol blocked the anchorage-independent growth of GBM cells. Moreover, irinotecan exhibited a similar inhibitory effect on colony formation of these examined GBM cells (Figure 2C). Our data suggest that xanthohumol decreases the cell growth of GBM cells in a dose- and time-dependent manner.

**Xanthohumol attenuates HK2 protein level and glycolysis in GBM cells**. Previous studies have demonstrated that the first rate-limiting enzyme of glycolysis, HK2, is deregulated in the tumorigenesis of GBM. We then examined the effect of xanthohumol on glycolysis and HK2 protein expression in human GBM cells. As data shown in Figure 2, xanthohumol significantly down-regulated HK2 expression in U87 (Figure 2A, left), T98G (Figure 2B, left) and LN229 (Figure 2C, left) cells. Importantly, accompanied by the decrease of HK2, xanthohumol suppressed the glucose uptake (Figure 2, middle) and lactate production (Figure 2, right) of GMB cells. U87 (Figure 2A, middle), T98G (Figure 2B, middle) and LN229 (Figure 2C, middle) cells treated with xanthohumol (10 μM) showed lower glucose uptake and lactate production rate (Figure 2A, B and C, right) than the control group. We further determined the mitochondrial outer membrane localization of HK2 in xanthohumol treated GBM cells. Our data showed that the HK2 protein levels in the mitochondrial fractions were decreased in U87, T98G, and LN229 cells (Figure 2D) after xanthohumol treatment, 5 μM of xanthohumol blocked HK2 mitochondrial localization. The mitochondrial HK2 interacts with the VDAC to inhibit mitochondrial apoptosis via suppressing of the release of cytochrome c. In Figure 2D, the western blot data showed that the biomarkers of apoptosis, cleaved-PARP and -caspase 3, were dramatically up-regulated in xanthohumol treatment groups in U87 (Figure 2D, left), T98G (Figure 2D, middle) and LN229 (Figure 2D, right) cells. These results indicate that xanthohumol suppresses tumor cell glycolysis and the decrease of HK2 mitochondrial localization.

### Xanthohumol promotes the proteasome-mediated degradation of c-Myc in GBM cells

**Figure 3:**
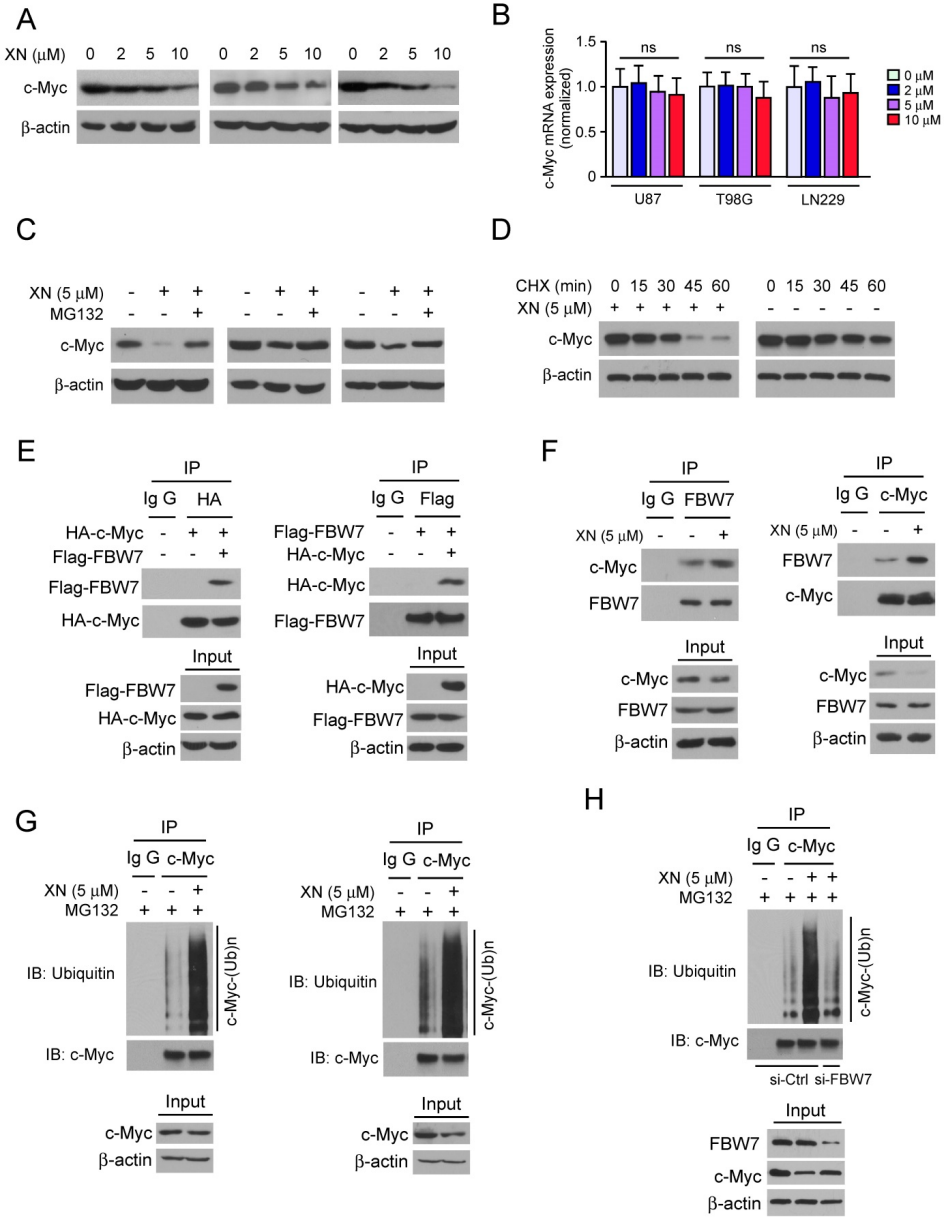
Xanthohumol promotes the proteasome-mediated degradation of c-Myc in GBM cells. A, xanthohumol decreases the protein levels of c-Myc in GBM cells. U87 (left), T98G (middle), and LN229 (right) cells were treated with xanthohumol for 24 h. Western blot was conducted to detect target proteins as indicated. B, the effect of xanthohumol on c-Myc mRNA expression in GBM cells, U87 (left), T98G (middle) and LN229 (right) cells were treated with xanthohumol for 24 h, qPCR was conducted to determine the mRNA level of c-Myc. C, U87 (left), T98G (middle) and LN229 (right) cells were treated with xanthohumol for 24 h as indicated, 20 μM of MG132 was added to the cell culture medium and incubated for another 6 h. The whole-cell lysate was extracted, and western blot was conducted to detect the target proteins. D, The U87 cells were treated with 20 μM CHX at multiple time points, whole-cell lysate was extracted, and western blot was conducted to detect the target proteins. E, HA-c-Myc, and Flag-FBW7 plasmids were co-transfected into 293T cells for 48 h. The whole-cell extract was incubated with the HA antibody (left) or Flag antibody (right) for co-immunoprecipitation. Western blot was conducted to detect the target proteins. F, U87 cells were treated with or without xanthohumol, whole-cell lysate was extracted and incubated with the FBW7 antibody (left) or c-Myc antibody (right) for co-Immunoprecipitation. Western blot was conducted to detect the target proteins. G, the U87 (left) and T89G (right) cells were treated with xanthohumol for 24 h, then incubated with MG132 for another 6 h, whole-cell lysate was extracted and incubated with c-Myc antibody at cold room overnight, western blot was used to determine the ubiquitination of c-Myc. H, U87 cells were transfected with si-ctrl or si-FBW7 siRNA for 12 h, the xanthohumol was added to the medium and cultured for 24 h, followed by incubation with MG132 for 6 h, co-immunoprecipitation and western blot were used to detect the ubiquitination of c-Myc.

To further determine whether c-Myc is required for xanthohumol-mediated down-regulation of glycolysis, we performed qRT-PCR and immunoblot analysis to examine the protein level and messenger RNA level of c-Myc in xanthohumol treated GBM cells. The result showed that xanthohumol dramatically decreased the protein expression of c-Myc (Figure 3A), whereas no significant difference in the mRNA level (Figure 3B) in U87, T98G, and LN229 cells.

Importantly, the proteasome inhibitor, MG132, strikingly rescued xanthohumol-induced down-regulation of c-Myc protein (Figure 3C). Exposure to xanthohumol resulted in the destabilization of c-Myc, the half-life of c-Myc protein was decreased in xanthohumol treated group (Figure 3D). These results suggested that the degradation of c-Myc was involved in xanthohumol-induced down-gradation of c-Myc in GBM cells. The GSK3β-FBW7 axis is required for ubiquitination-mediated degradation of c-Myc 34. Here, we found that xanthohumol promoted the interaction between c-Myc and FBW7 in 293T cells (Figure 3E). Moreover, in U87 cells, xanthohumol down-regulated c-Myc expression, and the endogenous binding activity was also enhanced after xanthohumol treatment, whereas xanthohumol had no significant effect on the expression of FBW7 (Figure 3F). We further determined the ubiquitination of c-Myc in xanthohumol-treated U87 and T98G cells using the CO-IP method.

We found that xanthohumol significantly promoted the ubiquitination of c-Myc in both U87 (Figure 3G, left) and T98G cells (Figure 3G, right). In order to further determine whether xanthohumol-induced up-regulation of c-Myc ubiquitination is dependent on E3 ligase FBW7, we knocked down the gene expression of FBW7 via siRNA in U87 cells. The western blot data showed that knocking down of FBW7 suppressed xanthohumol-induced c-Myc ubiquitination (Figure 3H). Taken together, these data indicate that xanthohumol promotes the ubiquitination-mediated degradation of c-Myc in GBM cells, and E3 ligase FBW7 was required for this regulation.

**Figure 4:**
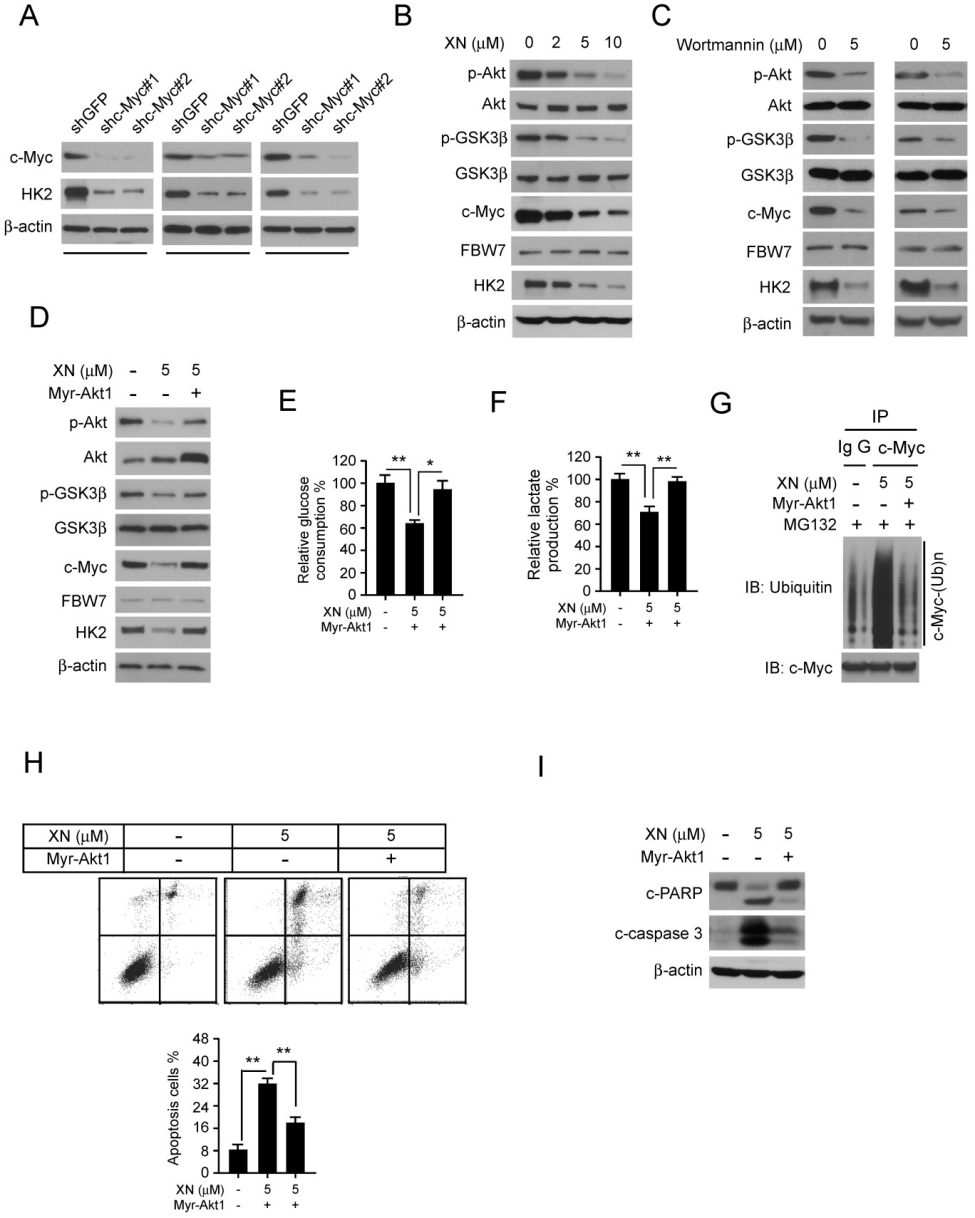
Down-regulation of Akt-GSK3β-FBW7 signaling axis results in the destabilization of c-Myc and inhibition of glycolysis. A, Stable knockdown of c-Myc in U87 (left), T98G (middle), and LN229 (right) cells were performed. Western blotting was conducted to detect the target proteins. B, U87 cells were treated with xanthohumol for 24 h. Western blotting was performed. C, U87 (left) and T98G (right) cells were treated with wortmannin for 24 h, western blotting was performed. D, E, and F, the Myr-Akt1 or vehicle plasmid was transfected into U87 cells, followed by treated with xanthohumol for another 24 h as indicated. Western blot analysis was conducted to detect the protein expression levels (D), and glucose consumption (E) and lactate production (F) were examined in these cells. Asterisk, significant suppression (*p<0.05, **p<0.01) of glycolysis between xanthohumol and DMSO treated group or Myr-Akt1 transfected group. G, the Myr-Akt1 or vehicle plasmid was transfected into U87 cells. These cells were treated with xanthohumol for another 24 h as indicated, followed by incubated with MG132 for 6 h, immunoprecipitation and western blot were used to detect the ubiquitination of c-Myc. H and I, the Myr-Akt1 or vehicle plasmid, was transfected into U87 cells. These cells were treated with xanthohumol for another 24 h. Flow cytometry was used for detecting of apoptosis (H), western blot analysis was performed to detect the expression levels cleaved -PARP and -caspase 3 (I).

**Down-regulation of Akt-GSK3β-FBW7 signaling axis promoted the destabilization of c-Myc.** In order to further demonstrate that c-Myc is essential for HK2 expression in GBM cells, the c-Myc knockdown stable cell lines in U87, T98G, and LN229 were constructed. The western blot data showed that inhibition of c-Myc expression significantly decreased HK2 protein level (Figure 4A). As the results shown in Figure 3, xanthohumol promoted the interaction between FBW7 and c-Myc and up-regulated c-Myc ubiquitination. We next examined the inhibitory effect of xanthohumol on the Akt-GSK3β-FBW7 axis in GBM cells. Results showed that the pAkt (Ser473) and pGSK3β (Ser9), as well as the expression of c-Myc and HK2, were down-regulated in xanthohumol treated U87 cells (Figure 4B). Moreover, the PI3-K/Akt pathway inhibitor, wortmannin, dramatically suppressed the pAkt, pGSK3β, and the expression of c-Myc and HK2 in U87 and T98G cells (Figure 4C). To elucidate the crucial role of Akt in xanthohumol-mediated glycolysis suppression in GBM cells, transient transfection of constitutively activated Akt (Myr-Akt1) was performed in U87 cell. The result showed that xanthohumol-mediated decrease of c-Myc and HK2 were significantly attenuated in Myr-Akt1 transfected group (Figure 4D). Importantly, xanthohumol-mediated reduction of glucose consumption (Figure 4E) and lactate production (Figure 4F) was substantially rescued with the increase of Akt activity. We next determined the ubiquitination of c-Myc in Myr-Akt1 transfected cells, along with the up-regulation of c-Myc (Figure 4D), the immunoblot result indicated that promotion of Akt activity significantly restricted xanthohumol-induced c-Myc ubiquitination (Figure 4G). Additionally, the flow cytometry data showed that transfection of myr-Akt1rescued xanthohumol-induced apoptosis in U87 (Figure 4G) cells, and decreased the expression of cleaved-caspase 3 and -PARP (Figure 4H). Overall, our results imply that suppression of Akt-GSK3β-FBW7 signaling pathway results in down-regulation of c-Myc expression and glycolysis in GBM cells.

**Figure 5:**
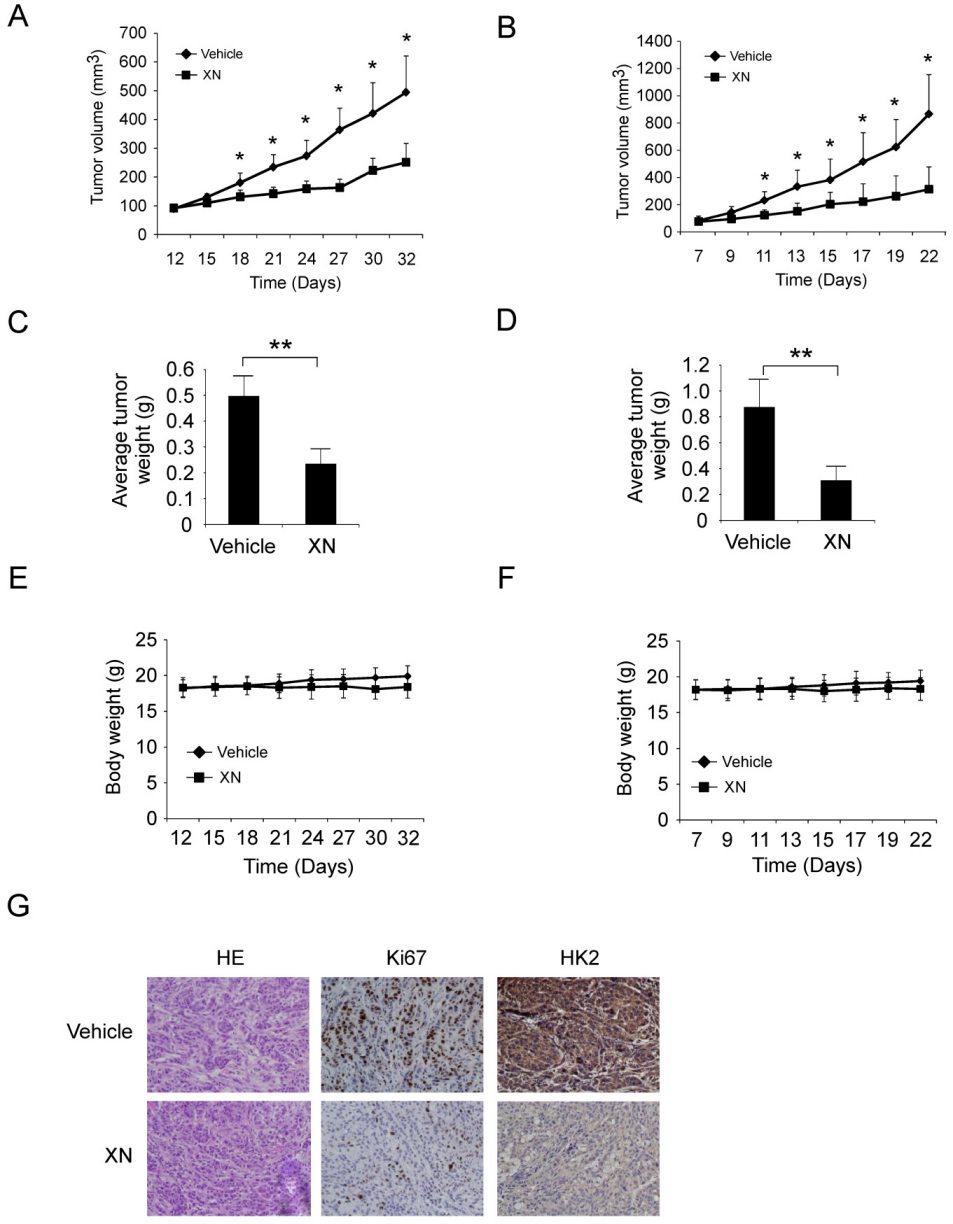
Xanthohumol inhibits tumor growth in xenograft mouse model. A and B, LN229 (A) and U87 (B) cells were subcutaneously injected into the flank of mice, after tumor volumes reached at 100 mm^3^, the mice were treated with vehicle or xanthohumol, tumor volumes were measured every two or three days. C and D, The average tumor weight from LN229 (C) and U87 (D) xenograft models treated with vehicle or xanthohumol were measured. E and F, During the treatment period, the bodyweight of LN229 (E) and U87 (F) xenograft mice was measured twice a week to determine the effect of xanthohumol. For A, B, C, and D, data are shown as mean values ± S.D. obtained from 5 mice in each group. G, Immunohistochemical staining examination of Ki67 and HK2 expression in tumor sections from vehicle- or xanthohumol-treated group mice.

**Xanthohumol suppresses GBM tumor cells growth *in vivo*.** To investigate the anti-tumor effect of xanthohumol *in vivo*, we performed the xenograft mouse model using the LN229 and U87 cells. The LN229 and U87 cells were injected (s.c.) into the right flank of six-week-old female athymic nude mice. Xanthohumol treatment (10 mg/kg/day) was initiated when the size of the tumor reached around 100 mm^3^. Results indicated that xanthohumol significantly reduced tumor size in LN229 *in vivo*. Results showed that the average tumor size of the vehicle-treated control group had reached around 500 mm^3^, however, the average tumor size of the xanthohumol-treated group was about 250 mm^3^ (Figure 5A, Supplementary Figure 2A). The average tumor weights of the vehicle-treated group and xanthohumol-treated group were 0.49 ± 0.08 g and 0.23 ± 0.06 g, respectively (Figure 5C). The similar results also obtained in U87 xenograft mouse model, treatment with xanthohumol significantly suppressed tumor growth *in vivo* (Figure 5B and 5D, and Supplementary Figure 2B). The mice body weight of both xanthohumol- and the vehicle-treated group had no significant difference (Figure 5E and 5F). IHC data suggested that the HK2 and Ki-67 were suppressed in the xanthohumol-treated tumor tissues (Figure 5G). We further compared the anti-tumor effect of xanthohumol with irinotecan *in vivo*. Our data demonstrated that both xanthohumol and irinotecan suppressed tumor growth significantly. However, irinotecan exhibited a much stronger inhibitory effect than that of xanthohumol (Supplementary Figure 2C-2F). Results indicated that the average tumor size of the vehicle-treated group was 612 ± 145 mm^3^, whereas average tumor size of the xanthohumol-treated group and the irinotecan-treated group was 364 ± 86 mm^3^ and 244 ± 51 mm^3^, respectively (Supplementary Figure 2D). These results indicated that xanthohumol inhibits GBM tumor cells growth in vivo.

## Discussion

Metabolic reprogramming, especially deregulation of glycolysis, is one of the hallmarks of human cancer. Recently studies have demonstrated that deregulation of glycolysis confers several tumorigenic advantages to GBMs, including facilitation of proliferation, migration, and invasion. Consequently, the accumulation of lactate forms a specific microenvironmental condition to promote tumor cell invasion and metastasis 35-39. The glycolytic enzymes, including the glucose transporter 1 (Glut1), HK2 and pyruvate kinase 2 (PKM2), are deregulated in human GBM cells and play exclusive effect in the tumorigenesis of GBM 36, 40-42. Thus, targeting the metabolic signaling and the rate-limiting enzymes could be an optional strategy for GBM treatment.

HK2 is required for the aberrant aerobic glycolysis in human glioblastoma multiforme, and overexpression of HK2 promotes tumor growth and progression 36, 43. Here, with xanthohumol treatment, the expression of HK-2, as well as glycolysis in GBM cells, were significantly decreased. This result further confirmed that HK2 is required for the maintaining of the malignant phenotype of GBM cells 36, 43. Localization to the mitochondria enhances HK-2 the advantage to avoid the inhibition of the glycolytic product, and further promote the ATP generation. Moreover, localization to the mitochondria and forms the complex with VDAC is required for HK2 to inhibit apoptosis 44-46. Targeting HK2 increases the sensitivity to radio/chemotherapy in GBM and promotes apoptosis 43. In the present study, the results showed that xanthohumol decreased the mitochondrial protein levels of HK2 and increased apoptosis in GBM cells (Figure 2D), which implied that xanthohumol-induced apoptosis might mainly attribute to the reduction of HK2.

The regulation of HK2 expression in tumor cells is complicated. It was evidenced that the enzymes in the glycolytic pathway were always regulated by oncoprotein c-Myc and HIF-1α 47-50. Further investigations revealed that microRNAs, including miR-143, miR-155, miR-218, and lincRNA-RoR 51-54 was also involved in the modulation of HK2 expression. Here, we showed that xanthohumol inhibited the protein level of c-Myc (Figure 3A) and shRNA-mediated gene silence of c-Myc substantially suppressed HK2 expression (Figure 4A). These results suggested that xanthohumol-induced down-regulation of HK2 may-dependent on inhibition of c-Myc. The PI3-K/Akt signaling pathway plays a central role in the regulation of metabolic reprogramming in human cancers. Evidence from the transgenic mouse has shown that overexpression of PTEN inhibited the expression of c-Myc and mimicked an 'anti-Warburg effect state', which suggests the cross-talking between PI3-K/Akt and c-Myc manufacturing the balance in control the cancer cell metabolic reprogramming 55, 56. Additionally, c-Myc was phosphorylated by GSK3β, and finally be recognized by FBW7 for ubiquitination-mediated degradation 34, 57, 58. Clinical tissues and TCGA database analysis revealed that FBW7 expression was correlated inversely with glioma histology and positively with patient survival time 59. Our results showed that xanthohumol-induced suppression of c-Myc could be rescued by proteasome inhibitor MG132, and treatment with xanthohumol promoted FBW7-mediated c-Myc ubiquitination (Figure 3). These results implied that the FBW7 mediated degradation of c-Myc was involved in xanthohumol-mediated c-Myc inhibition. Moreover, as one of the essential downstream target, the activation of GSK3β was regulated by Akt. Our result showed that xanthohumol attenuated the activation of the Akt pathway, and increased the activity of the GSK3β-FBW7 signaling axis and promoted c-Myc degradation (Figure 4B). Importantly, overexpression of Myr-Akt1 rescued the impaired glycolysis, c-Myc ubiquitination, and apoptosis in U87 cell (Figure 4D-I), which further confirm that the Akt-GSK3β-FBW7-c-Myc signaling axis was involved in xanthohumol-mediated glycolysis suppression.

Our result showed that treatment with xanthohumol didn't cause loss of body weight significantly (Figure 5E and F, Figure S2 F), suggesting that this compound is well-tolerated *in vivo*. This data consistent with previous findings that xanthohumol exhibited very low or no toxicity in normal cells, including human lung fibroblast cells, hepatocytes, oligodendroglia-derived cells, and skin fibroblasts 60. Moreover, daily administration of xanthohumol did not cause any noticeable sign of toxicity in the liver, exocrine pancreas, bone marrow, and kidneys in mice and female SD rats 60. Even the current literatures provide supporting evidence for the use of xanthohumol as an anticancer agent, but there are several barriers from basic research to clinical practice, including the discovery of novel target proteins, overcome low bioavailability and low extractive yield 60. Recently, human pharmacokinetics of xanthohumol showed a linear relationship between total xanthohumol plasma concentration (Cmax) and dose. Moreover, males tended to have lower Cmax values (83± 22 μg/L) than females (178 ± 34 μg/L) with a single oral dose of 180 mg, and the Tmax values for males and females were 3.1 ± 0.9 h and 1.1 ± 0.1, respectively. The human clearance rate for a single oral 180 mg xanthohumol dose was 3.2 L/kg×h^61^. However, the circulating levels of free xanthohumol were found to be very low compared to conjugate levels (< 4 %) 61. Pang *et al.* found that over 90% of the intracellular xanthohumol was localized in the cytosol and bound to cellular proteins, which may be the major factor responsible for poor oral bioavailability *in vivo* 62. Thus, chemical structure modification or optimization of the compound properties to attenuate the binding activity of xanthohumol to cytosolic proteins could be a promising approach to enhance the bioavailability of xanthohumol.

## Conclusion

In summary, this study indicated that inhibition of glycolysis is required for xanthohumol-mediated anti-tumor activity. Suppression of the Akt-GSK3β-FBW7-c-Myc signaling pathway and disruption of the stability of c-Myc protein could be one of the major underlying mechanisms for xanthohumol-induced anti-GBM effect. This study suggested that xanthohumol may be a promising new anti-tumor agent which deserves further study.

## Supplementary Material

Supplementary figures.
