# Supplementary material for: Xanthohumol suppresses glioblastoma via modulation of Hexokinase 2 -mediated glycolysis
Source: J Cancer. 2020 Apr 6;11(14):4047–58. doi: 10.7150/jca.33045 (PMC7196271; doi:10.7150/jca.33045)

Supplementary Figure 1. Inhibitory effects of xanthohumol and irinotecan on the growth of GBM cells. The U87 (A), T98G (B), and LN229 (C) cells were treated with DMSO, xanthohumol, or irinotecan for 72 h. Cell proliferation assay was performed as described in the "Material and Methods". Data shown are the proliferation ability of human GBM cells treated with xanthohumol compared with the irinotecan -treated group. Iri, irinotecan. Asterisk, significant suppression (*p<0.05, **p<0.01) of proliferation by irinotecan.

Supplementary Figure 2. Xanthohumol and irinotecan inhibit tumor growth in xenograft mouse model. A and B, LN229 (A) and U87 (B) cells were injected into nude mice to create xenografts, and mice were treated with vehicle control or xanthohumol, and tumors were dissected. C-F, LN229 cells were injected into nude mice to create xenografts, and mice were treated with vehicle control, xanthohumol, or irinotecan. Tumor size was monitored (**C**). Tumors were dissected (**D**) and weighed (**E**), and mice body weight was measured (F). Data are shown as mean values ± S.D. obtained from 5 mice in each group. *$p < 0.05$, **$p < 0.01$.

Supplementary Figure 1

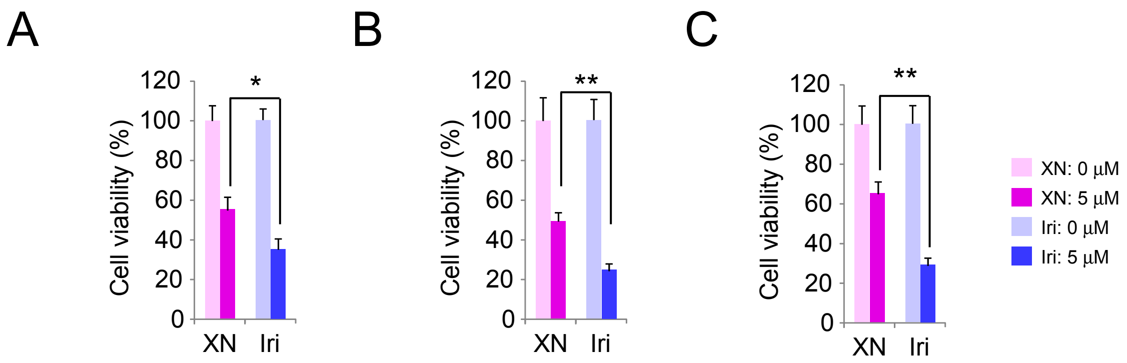

Supplementary Figure 2

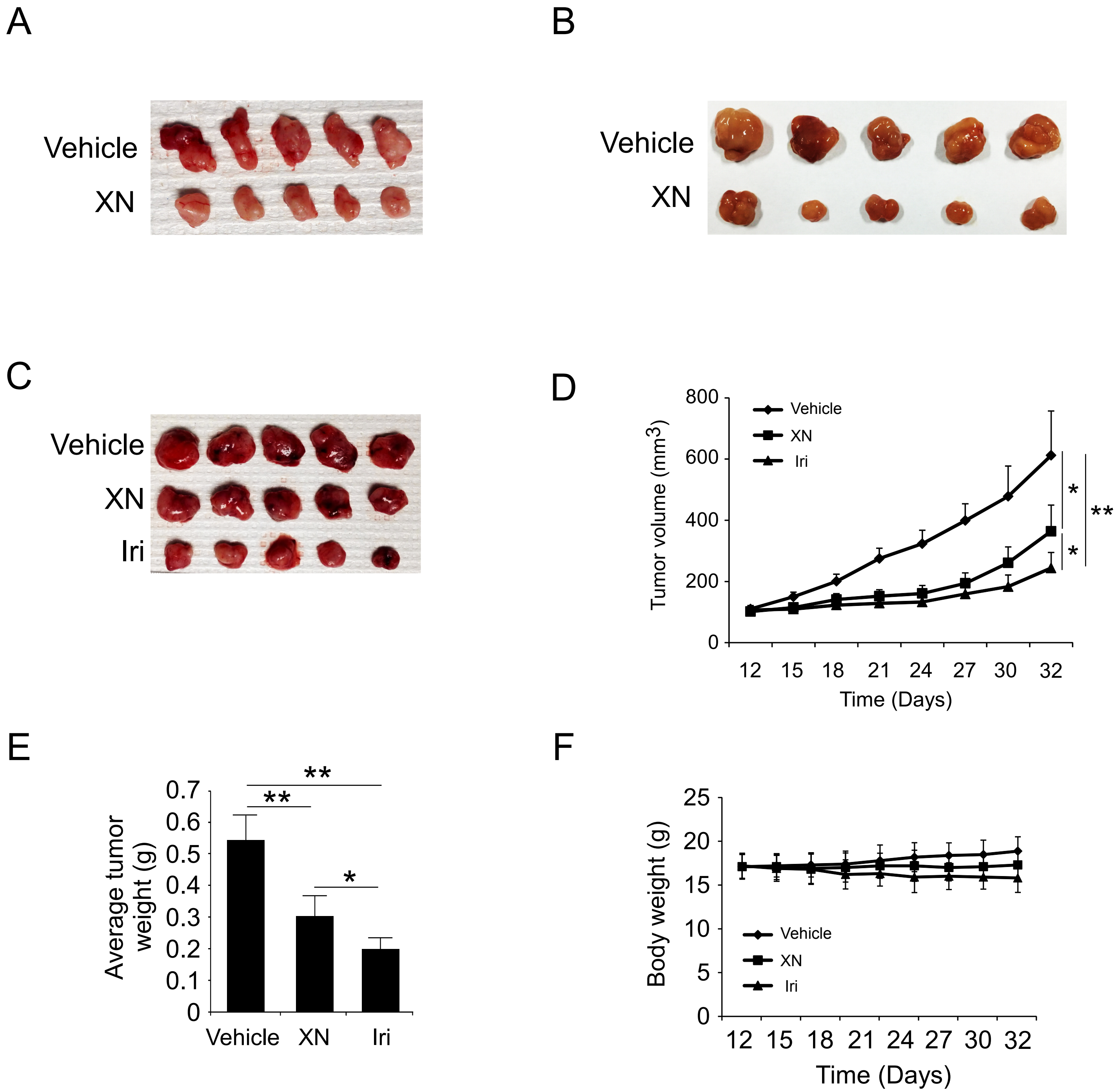

Supplement: Supplementary file 1 — Supplementary figures. [file jcav11p4047s1.pdf]
